# The contributions of surface features and contour shapes to object slant perception

**DOI:** 10.1177/20416695231160402

**Published:** 2023-03-09

**Authors:** Ryosuke Niimi

**Affiliations:** Faculty of Humanities, Niigata University, Niigata, Japan

**Keywords:** visual illusion, slant perception, Oppel-Kundt illusion, horizontal-vertical illusion

## Abstract

Humans perceive 3D shapes even from 2D images. A slant can be perceived from images of slanted rectangular objects, which include texture gradients and linear perspective contours. How does the visual system integrate and utilize these pictorial depth cues? A new visual illusion that provides some insights into this issue was examined. A box-like object with disk figures drawn on its upper surface was rendered in a linear perspective image. The length of the object's upper surface side line was overestimated, probably due to the foreshortened disks serving as slant cues. This illusory effect occurred even when observers estimated the line length on the image plane, suggesting that the slant perception from the disks was mandatory. Five experiments revealed that multiple depth cues were utilized for the slant perception; the aspect ratio of the disks, texture gradients, trapezium/parallelogram contours, and the side surfaces of the box-like object. However, foreshortened disks outside the object were not utilized as depth cues. These results suggested that various depth cues belonging to the target object are integrated for the slant perception.

In today's society, where various media abound, visual depth perception from two-dimensional images is quite common. Humans perceive depth even from two-dimensional images on paper or screens (Vishwanath, 2014). This ability is based on pictorial depth cues, including linear perspective, texture gradients, shading, and occlusions. Although individual depth cues are sufficient to produce substantial depth perception, they often provide ambiguous depth information and may contradict each other. The issue that arises here is the mechanism for integrating multiple depth cues.

How does the visual system integrate pictorial depth cues when observing an image? Research on slant perception has involved this issue (for review, see Perrone, 1982; Saunders & Backus, 2006a). In general, visual judgments on object slant are often inaccurate (Koenderink et al., 1992; Oomes & Dijkstra, 2002; Rosinski et al., 1980). Determining a slant angle from a single depth cue is usually tricky. For instance, a 2D projection of a slanted rectangular object yields a trapezium contour shape. Slant estimation from such a perspective image is an ill-posed problem and, therefore, inaccurate (Saunders & Backus, 2006a; Smith, 1967). If we posit constraints that the 3D object is rectangular (i.e., all the internal angles are 90°) and placed on the ground plane, and if the retinal size of the image is fixed, the object slant becomes computationally unambiguous. However, in our daily experiences in which we observe perspective images on papers and screens, the retinal size of the image is not stable, and slant estimation remains ambiguous (see Saunders & Backus, 2007). Gibson (1950) demonstrated the inaccuracy of slant perception from texture gradients. He presented images of texture gradients on a frontoparallel screen and asked the observers to report the perceived slant by adjusting a movable surface with their palms. The observers underestimated the slant. Namely, the perceived slant, on average, was biased toward the frontoparallel plane (0° slant). Gibson interpreted this “frontal tendency” as a compromise of the stimulus textures’ slant and the screen surface's frontal impression. Presenting texture gradients and trapezium contours simultaneously, however, such inaccuracy and bias in slant perception are mitigated to some extent (Saunders & Backus, 2006a; Zimmerman et al., 1995). Pictorial depth cues other than contours and texture gradients are likely integrated as well.

Niimi (2012) reported a new illusory figure (Figure 1A) that suggests a different integration of pictorial depth cues. In this figure, the side line (a) appears longer than the side line of another object (b), although they are, in reality, the same in length. The contour shape of the right-side object is a mirror reversal of that of the left-side one. The critical factor in the illusory effect is the surface figure of the objects. These figures were taken from a tile in the game of Mahjong, played in China and other regions. Figure 1A was created using 3D graphic software. As shown in Figure 1B, the original surface image of the Mahjong tile was pasted on the virtual square object so that the texture would fit the square contour. The virtual camera then rendered the two objects into the image shown in Figure 1A, consistent with a linear perspective.

**Figure 1.**
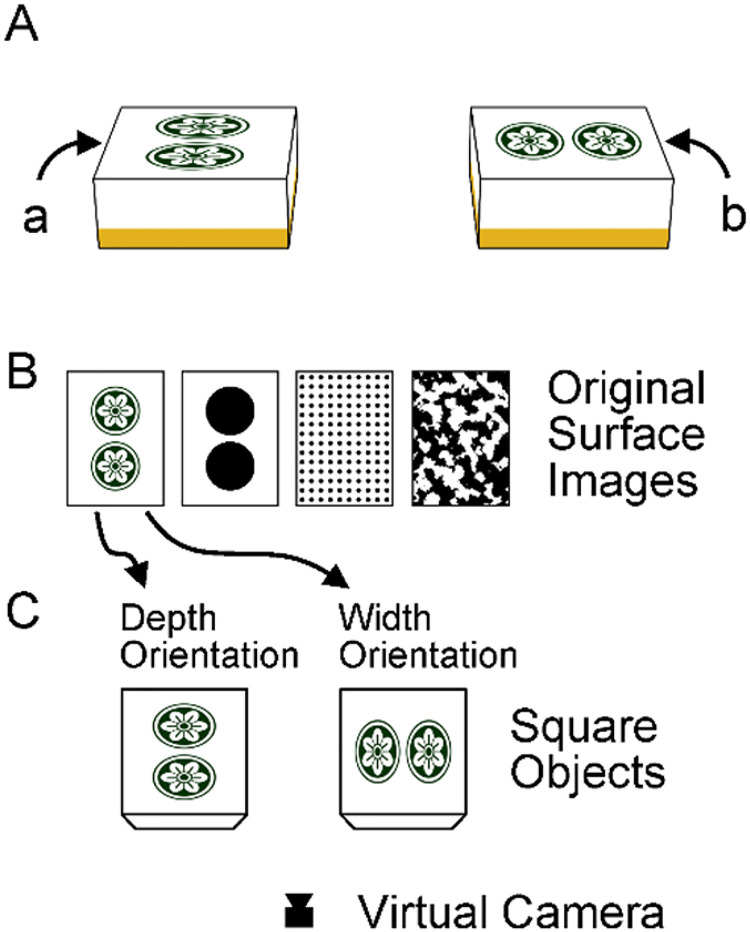
(A) The original illusion figure by Niimi (2012). The side line (a) appears longer than (b), but is the same length. (B) The surface images used in Figure 1A and in the stimuli of Experiments 1 and 2. (C) The image of Figure 1A was generated by 3D graphic software. The original images in Figure 1B were pasted on the upper surfaces of the virtual objects so that the images fit the square shape. The objects were then rendered into the stimulus image through the virtual camera.

The cursory observation in Figure 1A suggests that the illusion results from slant perception. Because the left-object surface figure is more foreshortened than the right-object, we perceive more slant for the upper surface of the left object, resulting in an impression that the surface is a depth-long rectangle. Such a 3D perception may be mandatory and thus distort the judgment of the 2D line length (i.e., the length on the image plane).

What is new about this illusion? Many visual illusions, including the Müller-Lyer and Ponzo illusions, have been explained by depth perception and shape constancy scaling (Gregory, 1963), although there are arguments against this theory (DeLucia & Hochberg, 1991; Newman & Newman, 1974; Todorović, 2020). In addition, one might point out that this illusion is related to the Shepard illusion (Shepard, 1981) and Sander's illusion (Robinson, 1998, pp. 24–26), both of which perceive line length as being affected by parallelogram contours. However, these illusion figures do not contain surface figures. The illusion of Figure 1A may provide new evidence of depth-related visual illusion and new insights into integrating depth cues from surface features and contour shapes.

Figure 1A contains multiple depth cues. The critical cue for the illusory effect should be the foreshortened surface figures, or more precisely, the aspect ratio and arrangement of the disk shapes. The aspect ratio of foreshortened disks is a pictorial cue for local slant (Stevens, 1981; Todd & Thaler, 2010). However, the object contour alone causes a clear perception of slant as well. Parallelogram and trapezium contours often create the perception of rectangular objects foreshortened in depth, although the perceived slant is not always accurate (Saunders & Backus, 2006a; Smith, 1967). The 3D interpretation of contour shapes seems to be based on several pictorial cues, such as line convergence and skew symmetry (Saunders & Knill, 2001; Saunders & Backus, 2007). Consequently, trapezium/parallelogram shapes sometimes cause visual illusions related to shape constancy (Bressanelli & Massironi, 2007; Griffiths & Zaidi, 2000). In addition to the trapezium contours of the upper surfaces, Figure 1A objects contain side surfaces, which may serve as depth cue as well.

The current study examined how these multiple depth cues are utilized and integrated by assessing the illusory effect. The researcher manipulated and examined the effects of the disks, texture gradients, contour shapes, and side surfaces.

## Experiments 1A and 1B

**Figure 2.**
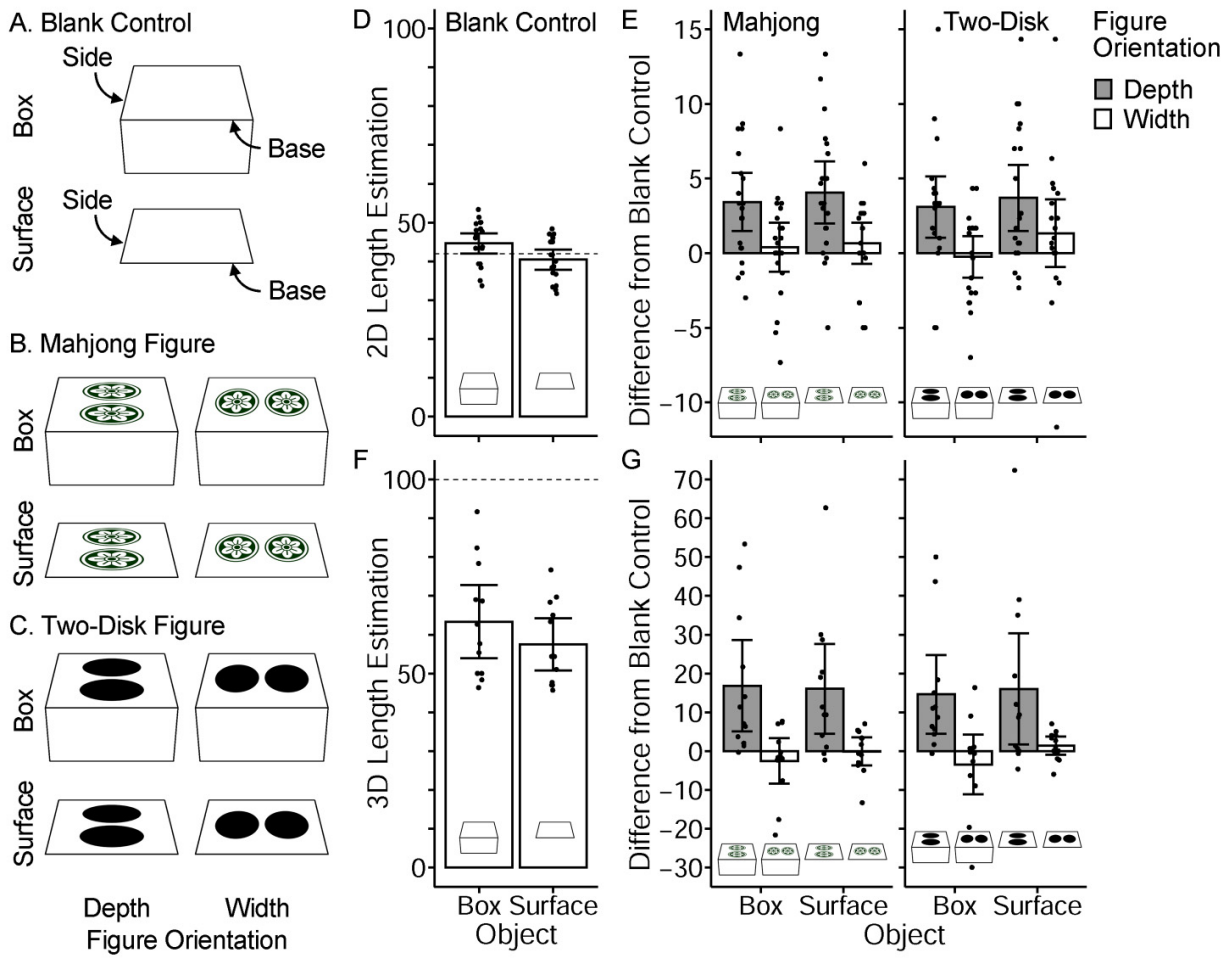
(A), (B), and (C) show the stimulus images of Experiments 1A and 1B. The task was to estimate the side length relative to the base length of 100. In Experiment 1A, participants were asked to estimate the length on the image plane (i.e., 2D length). In Experiment 2B, they were asked to imagine the 3D object depicted and estimate the length in terms of 3D space. (D) The estimated side length of the blank-control condition in Experiment 1A. The dotted line indicates the actual side line length (42% of the base line). (E) The results of stimuli with surface figures in Experiment 1A (difference from the corresponding blank-control conditions). (F) and (G) show the results of Experiment 1B. The dotted line indicates the actual 3D length of the side line. Error bars represent 95% CI of the means. Each dot represents the mean of each participant.

Experiment 1A quantitatively confirmed the illusory effect and examined the effects of surface figures and contours separately. The participants observed the stimulus images (Figures 2A to C) and estimated the length of the side line of the trapezium contour. Unlike in the original figure (Figure 1A), only one object was presented in each trial. The surface figures (disks) were absent in the blank control conditions (Figure 2A). Any effect of the surface figures was measured as the difference from the blank-control conditions. Experiment 1A also examined whether a box-like contour shape affects the illusion. The box-condition stimulus contained the side and upper surfaces, whereas the surface-condition stimulus contained only the upper surface. If the illusion is derived solely from the upper surfaces, the illusory effect should be observed equally for box and surface stimuli.

Experiment 1B adopted an identical set of stimulus images to those used in Experiment 1A. The only difference was in the task assigned. The participants estimated the 3D length of the side line rather than its 2D length on the image plane. This experiment was conducted to estimate the strength of the slant perception elicited by stimulus images. The author predicted that the 3D length estimation would be larger (i.e., larger angle of perceived slant) in Experiment 1B under the condition in which the illusory effect was observed in Experiment 1A.

### Method

#### Participants

Thirty-two individuals (20 in Experiment 1A and 12 in 1B; mean age: 22.8 years) participated in this research. All participants reported normal or corrected-to-normal vision. The Institutional Review Board of the Department of Psychology, The University of Tokyo, approved the procedures of all experiments reported in this paper.

#### Stimuli and Design

Stimuli were generated using 3D graphic software. The original surface images were 400 (W) × 560 (H) pixels (see Figure 1B). A square object was created in the virtual 3D space, and the original images were stretched and pasted onto the square object's upper surface so that the images filled the surface (Figure 1C). Objects with surface images were rendered into stimulus images using a virtual camera positioned to replicate roughly the participants’ viewpoint. At this setting, the simulated slant angle of the upper surface of the object was 60°. The background was uniformly white.

Ten unique stimulus images (i.e., ten conditions) were used (see Figures 2). Five surface figure conditions were used: blank control, Mahjong-tile figure (depth/width), and two-disk figure (depth/width). There were two object conditions: the box and surface. The base line (bottom) and the side line (Figure 2A) subtended 14.2° and 6.0° (520 and 218 pixels) on the screen, respectively. The side length was 42% of the base length.

#### Apparatus and Procedure

The stimuli were shown on a 24-inch liquid crystal display (LCD) screen (Dell 197 U2410). One object was presented in each trial. The stimulus image's position was randomly shifted from the display center in each trial to avoid comparison with the length of the previous-trial stimulus. Participants sat in front of the display, observing the stimuli at a distance of approximately 56 cm. Their heads were not fixed in place by any apparatus.

Before the experiment began, the participants were shown the blank-control stimuli printed on a piece of paper and were told that their task was to estimate the length of the side line compared to the base length of 100 (arbitrary unit). Experiment 1A emphasized that the task was to estimate the 2D length on the screen surface and not in 3D space. In Experiment 1B, however, the participants were asked to imagine the 3D shape and estimate the side length in the 3D space. Otherwise, the procedure was the same in Experiments 1A and 1B.

The participants were given a pen and an answer sheet. In each trial, a small trial number was shown in the upper-left corner of the display. The participants wrote their estimates of the side line length in the designated box on the answer sheet. The stimulus image was presented until the space key was pressed. Each participant performed 30 trials in random order, in which all stimulus images appeared three times each. The experiment took approximately 10 min.

### Results and Discussion

Figures 2D and 2E show the results of Experiment 1A. As seen in Figure 2D, the estimated side length in the blank control conditions was distributed around the actual 2D side length (42% of the base length), indicating that the participants performed the task well. Interestingly, the blank-control box condition yielded longer estimates (*M* = 44.7, *SD* = 5.63) than the surface condition (*M* = 40.4, *SD* = 5.57). A paired *t*-test confirmed this difference (*t*(19) = 4.59, *p* < .001, Cohen's *d* = 0.75). The side surface in the box increased the perceived side length, suggesting a stronger slant perception.

The mean estimated length of the blank-control condition was subtracted from the participant's mean estimated length for conditions with surface figures to quantify the effect of surface figures for each condition. Note that any results from the blank-control box (surface) condition were subtracted from those of the box (surface) condition. This procedure canceled out the difference between the box and surface conditions reported above.

The difference results are shown in Figure 2E. As indicated by the error bars (95% CI), surface figures in depth-orientation condition yielded positive values significantly different from zero, indicating that the figures yielded longer estimates than the blank-control conditions. The mean difference was 3.6 for depth-orientation conditions, which corresponded to 8.5% of the actual side length (42) and was significantly larger than zero (one-sample *t-*test, *t*(19)_ _= 4.28, *p* < .001). In contrast, the mean difference for width-orientation figures was not different from zero (*M* = 0.5, *t*(19) = 1.31, *p* = .204).

A three-way repeated measures analysis of variance (ANOVA) was conducted on the difference data (Figure 2E). The main effect of figure orientation (depth/width) was significant (*F*(1, 19) = 12.7, *p* = .001), while the main effects of figure type (Mahjong/two-disk) and object (box/surface) were not significant (*F*(1, 19) = 0.1, *p* = .707; *F*(1, 19) = 1.0, *p* = .337, respectively). No interaction effect was observed (*p*s > .1). The illusion was confirmed because the depth-orientation yielded significantly longer estimates than the width-orientation. This illusory effect was independent of figure type and object condition. In particular, the depth-orientation surface figures yielded significantly longer estimates than the blank-control conditions, suggesting that those figures provided a stronger slant perception.

Experiment 1B results were analyzed in the same manner. The estimated side lengths in the blank-control conditions (Figure 2F) were much longer than those in Experiment 1A (Figure 2D), which confirmed that the participants estimated 3D length, not 2D length, as instructed. However, the estimates were shorter than the veridical length (100) of the virtual square object, suggesting that the surface slant was underestimated.

Similar to the result of Experiment 1A, the blank-control box condition yielded significantly longer estimates (*M* = 63.4, *SD* = 14.82) than the blank-control surface condition (*M* = 57.5, *SD* = 10.63; paired *t*-test, *t*(11) = 2.20, *p* = .0497, *d* = 0.42). A three-way repeated measures ANOVA on the difference from blank control (Figure 2G) found only a significant main effect of figure orientation (*F*(1, 11) = 8.2, *p* = .015), suggesting that the participants perceived a larger slant angle for the surface with depth-orientation figure than the surface with width-orientation figure. The main effects of figure type and object were not significant (*F*(1, 11) = 0.4, *p* = .534; *F*(1, 11) = 0.6, *p* = .449, respectively). No interaction effect was observed (*p*s > .1).

To summarize, the illusion was quantitatively confirmed, and the illusory effect was comparable for the Mahjong and simplified two-disk figures. The elliptic contours, not the detailed pattern inside the Mahjong figure, served as depth cues. Additionally, the blank contour shape of the box object yielded a similar illusory effect. Both surface figures and contour shapes were used as depth cues. Since the pattern of results in Experiment 1B (3D length estimation) was equivalent to that in Experiment 1A, it was likely that the illusory effects reflected the perceived slant.

## Experiments 2A and 2B

**Figure 3.**
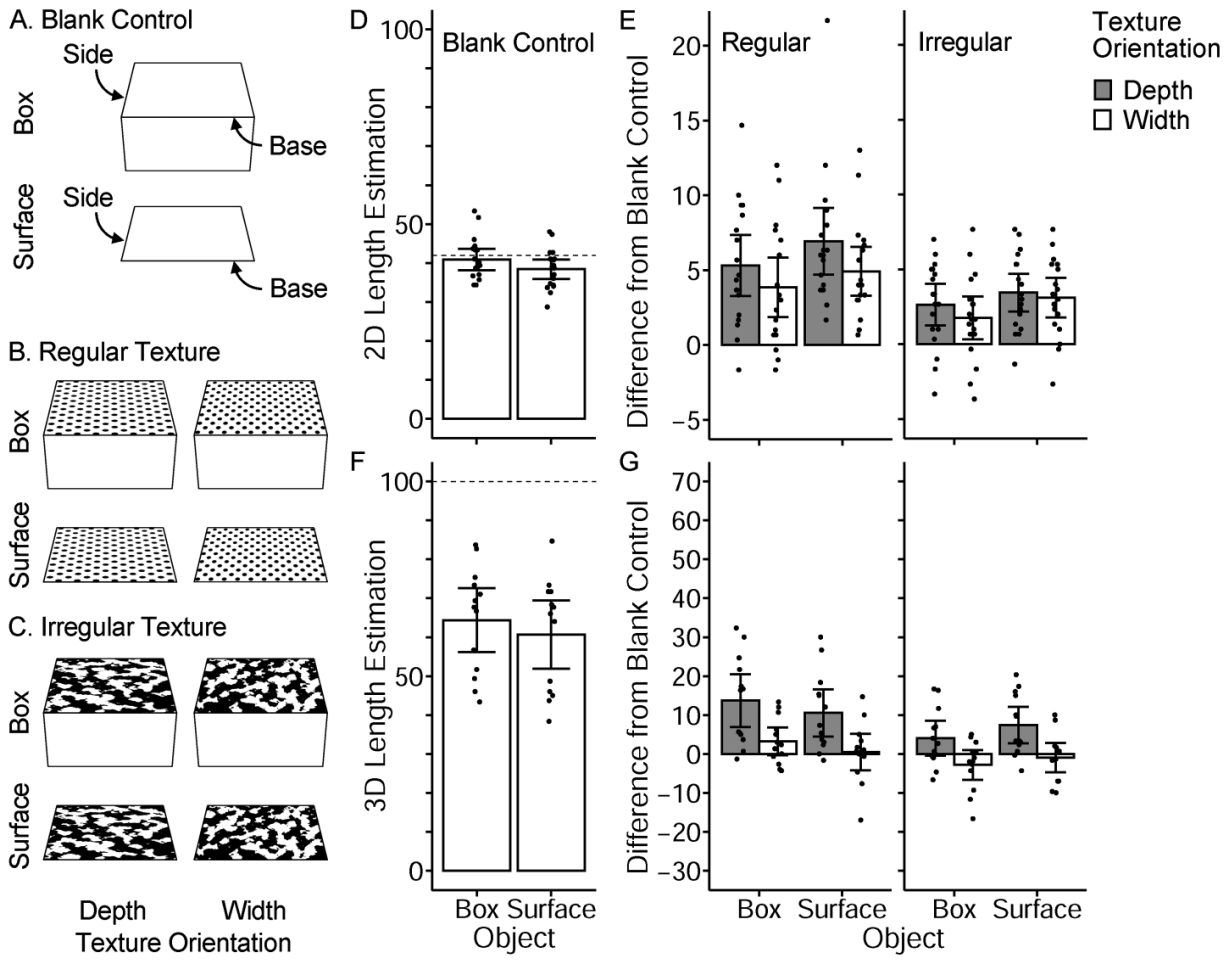
(A), (B), and (C) contain the stimuli of Experiments 2A and 2B. The task was identical to Experiments 1A and 1B (2D/3D length estimation of the side line). (D) and (E) indicate the results of Experiment 2A (2D length estimation). (F) and (G) contain the results of Experiment 2B (3D length estimation). The dotted lines indicate the actual 2D/3D length. Error bars represent 95% CI. Each dot represents the mean of each participant.

Experiments 1A and 1B suggested that the distorted disk figures on the surface and contours were used as depth cues and yielded the illusory perception of side line length. Experiments 2A and 2B examined whether the illusory effect would be observed in texture gradients (Figures 3B and 3C).

### Method

#### Participants

Thirty individuals (mean age = 22.2) volunteered. Eighteen participated in Experiment 2A and 13 in Experiment 2B, including one who participated in both. In addition, in advance, four participants from Experiment 2B participated in 1B. All participants reported normal or corrected-to-normal vision.

#### Stimuli and Procedure

The experimental design, task, procedure, and stimuli were the same as those in Experiments 1A and 1B, except for the surface images. Instead of the Mahjong figure and the two-disk figure, regular and irregular textures were adopted (Figures 3B and C). Since regular textures yield more accurate slant perception (i.e., less underestimation of slant) than irregular textures (Kraft & Winnick, 1967; Newman et al., 1973; Velisavljević & Elder, 2006), it was expected that the regular texture would cause a more illusory effect. As in Experiment 1, the original surface images were 400 (W) × 560 (H) pixels in size and were stretched and pasted on the virtual square texture using 3D graphic software. The irregular texture image was created by binarizing a random cloud texture image such that the areas of the black and white regions were equal.

In Experiment 2A, participants estimated the 2D side length in the same manner as in Experiment 1A. Experiment 2B participants estimated the 3D side length in the same manner as in Experiment 1B. Each experiment comprised 30 trials (10 unique stimulus images with three repeats each).

### Results and Discussion

The length estimations were analyzed in the same manner as in Experiment 1. Figures 3D and E display the results of Experiment 2A.

Again, the blank-control box condition yielded a significantly longer 2D estimation than the blank-control surface condition (*t*(17) = 2.96, *p* = .009, *d* = 0.46; Figure 3D). For regular and irregular texture conditions, differences from the blank-control conditions were analyzed (Figure 3E). Contrary to Experiment 1A, the difference values were significantly larger than zero irrespective of texture orientation. This result may be due to the Oppel-Kundt illusion (Robinson, 1998, pp. 49–52), in which the length of the region filled with small elements is overestimated compared to that of the blank region. A three-way ANOVA on the difference data revealed that the main effects of texture orientation (depth/width) and texture type (regular/irregular) were significant (*F*(1, 17) = 18.9, *p* < .001; *F*(1, 17) = 10.9, *p* = .004, respectively). The main effect of the object (box/surface) was not significant (*F*(1, 17) = 2.4, *p* = .142). However, the interaction between the texture orientation and texture type was significant (*F*(1, 17) = 6.3, *p* = .023). The simple main effect of texture orientation was significant for regular texture (*F*(1, 17) = 54.0, *p* < .001), but not for irregular texture (*F*(1, 17) = 2.6, *p* = .124). The regular texture yielded an illusory effect by texture orientation, whereas the irregular texture did not. No other interactions were statistically significant (*p*s > .1).

Experiment 2B results (3D length estimation) are shown in Figures 3F and 3G. In the blank-control condition, the difference between the box and surface stimuli was not statistically significant (*t*(12) = 1.65, *p* = .125, *d* = 0.26; Figure 3F), which was inconsistent with Experiment 1B blank-control conditions (Figure 2F). This failure in replication might be due to the modest sample sizes. The difference between regular/irregular texture and blank-control conditions (Figure 3G) was analyzed using a three-way ANOVA. The main effects of texture orientation (depth/width) and texture type (regular/irregular) were significant (*F*(1, 12) = 14.7, *p* = .002; *F*(1, 12) = 5.9, *p* = .032, respectively). The main effect of the object (box/surface) was not significant (*F* < 1). The interaction between texture type and object was significant (*F*(1, 12) = 7.6, *p* = .018), and the simple main effect of texture type was significant for the box condition (*F*(1, 12) = 9.2, *p* = .010), but not for the surface condition (*F*(1, 12) = 1.3, *p* = .276). As Figure 3G shows, the regular texture yielded a longer 3D length estimation in the box condition than the irregular texture. The interaction between texture orientation and texture type was not significant (*F*(1, 12) = 3.8, *p* = .076). Other interactions were not significant as well (*p*s > .1).

Overall, the regular texture yielded more effects than the irregular texture, both in the 2D task and 3D task. In addition, the illusory effect of texture orientation in the 2D length estimation was found only for the regular texture. This pattern of results seemed consistent with the previous reports of better slant perception (less slant underestimation) for regular textures.

It should be noted, however, that the illusory effect of the regular texture was much smaller than that in Experiment 1. The difference between depth- and width-orientation was relatively small in Experiment 2A (Figure 3E) compared to Experiment 1A (Figure 2E). The foreshortened disks in Experiment 1 might provide additional depth cues such as aspect ratio that were unavailable in the texture gradients.

## Experiment 3

**Figure 4.**
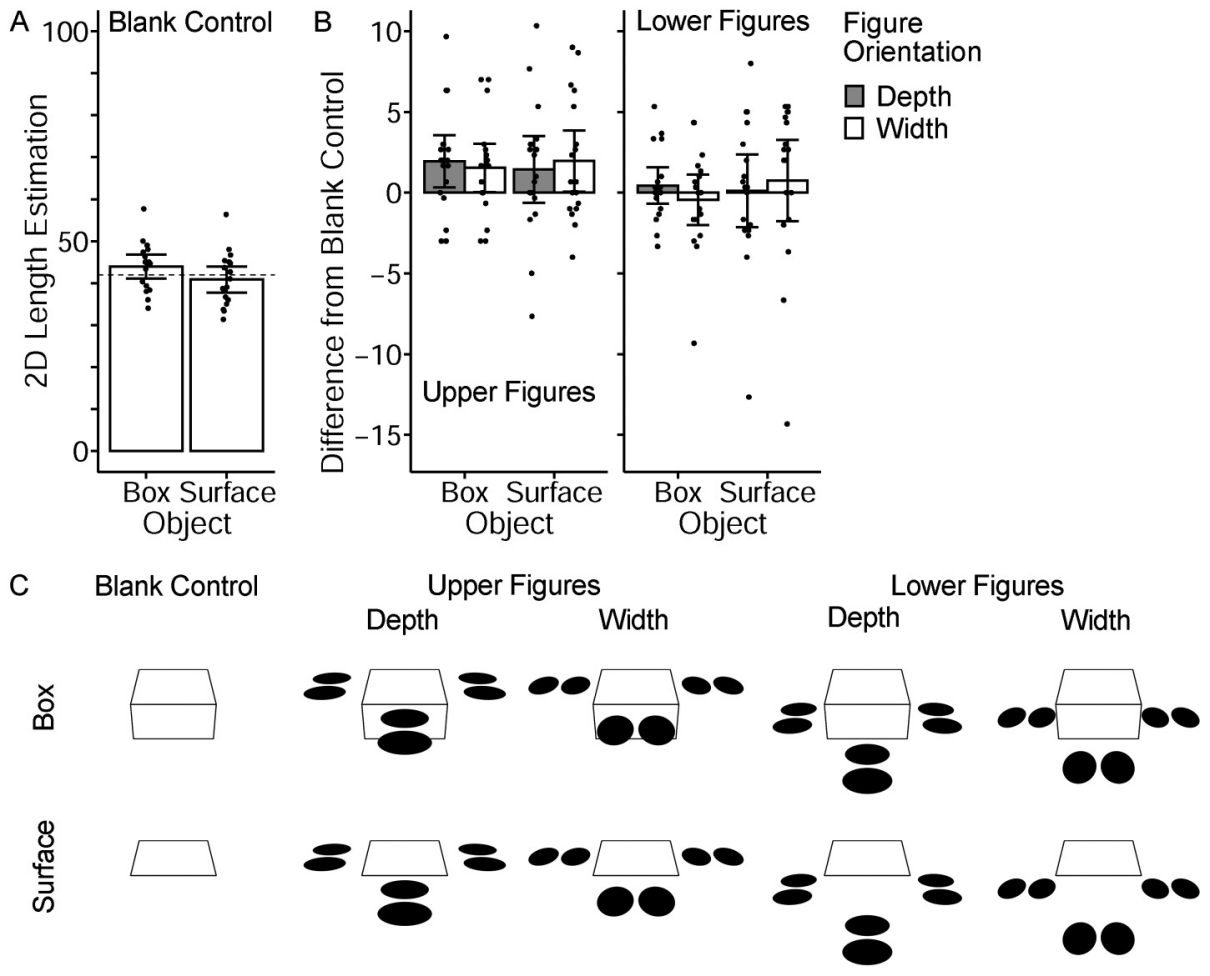
(A) and (B) indicate the results of Experiment 3, while (C) contains the stimuli of Experiment 3. The participants’ task was identical to Experiments 1A and 2A (2D length estimation). Error bars represent 95% CI. Each dot represents the mean of each participant.

In Experiment 3, the two-disk figure was placed outside the object rather than on it (Figure 4C). Such disks still cause slant perception of the global surface. If they cause an illusory effect, as in Experiment 1A, we could conclude that depth cues that do not belong to the object are also integrated for the slant perception of the object.

### Method

#### Participants

Eighteen individuals (mean age = 22.5) volunteered for this experiment. Only one of them had participated in Experiment 2B in advance, while none of the others had participated in any other experiments reported in this paper. All participants reported normal or corrected-to-normal vision.

#### Stimuli and Procedure

The experimental design, task, procedure, and stimuli were the same as those in Experiment 1A, except for the surface figures. Only a two-disk figure was used, placed outside the object (Figure 4C). Three two-disk figures were placed to surround the object, while the orientation of each figure was varied (depth/width). In addition, there were two levels of the height of the global surface where the figures were placed: upper and lower. The figures were at the same height as the upper surface (upper figure) or the bottom surface of the object (lower figure). As a result, there were five figure arrangements (figure height × figure orientation and blank control). Since there were two object conditions (box and surface), ten stimulus images were prepared, as shown in Figure 4C. They were rendered using 3D graphics software consistent with a linear perspective.

The task assigned was identical to that in Experiment 1A, namely, to estimate the 2D length of the side line of the object. Each participant performed 30 trials (10 stimulus images × 3 repetitions) in a randomized order.

### Results and Discussion

The mean estimated length in the blank-control condition was significantly longer for the box condition than for the surface condition (*t*(17)_ _= 3.86, *p* = .001, *d* = 0.51; Figure 4A), which was a replication of Experiments 1A and 2A.

The mean difference of 2D length estimation from the blank control was analyzed by a three-way ANOVA, which examined the within-participant factors of figure height (upper/lower), figure orientation (depth/width), and object (box/surface). As evinced in Figure 4B, the disk figures showed almost no effect. All main effects were not significant (figure height, *F*(1, 17) = 3.8, *p* = .070; figure orientation, *F*(1, 17) = 0.01, *p* = .913; object, *F*(1, 17) = 0.1, *p* = .806). Interaction effects were also not significant (*p*s > .1).

The disk figures outside the object did not cause an illusory effect. This result suggested that only surface figures belonging to the object were integrated to create a perception of the object's surface slant.

## Experiment 4

Experiments 1–3 showed that the foreshortened disks on the object surface were the primary source of the illusory effect of the original figure (Figure 1A). Which aspect of the disks caused this illusion? The purpose of Experiment 4 was to examine the effect of the disk aspect ratio (the disk height to disk width ratio). Small ratios, such as 0.2, would suggest a greater slant angle of the surface, resulting in perception of a longer side length than large ratios, such as 1.0.

Another purpose of Experiment 4 was to examine the effect of contour shapes. In Experiments 1–3, the contour shape of the upper surface of the object was trapezium. Experiment 4 adopted parallelogram and rectangle contours as well. In addition, it included an adjustment task. In Experiments 1–3, the participants directly estimated the side length, which was physically constant throughout the experiments. Such a task was sufficient for roughly examining whether or not the illusion occurred but was not suitable for quantifying the parametric effect of the disk aspect ratio (see Coren & Girgus, 1972). Experiment 4 participants were asked to adjust the side length of the contour shape so that it seemed equal to the bottom base line. The adjusted side length was then analyzed. A shorter adjusted length indicated that the participants perceived the side length to be longer.

### Method

#### Participants

Twenty individuals (mean age = 22.9) participated in Experiment 4. None of them participated in any of the other experiments described in this study. All participants reported normal or corrected-to-normal vision.

#### Stimuli and Design

**Figure 5.**
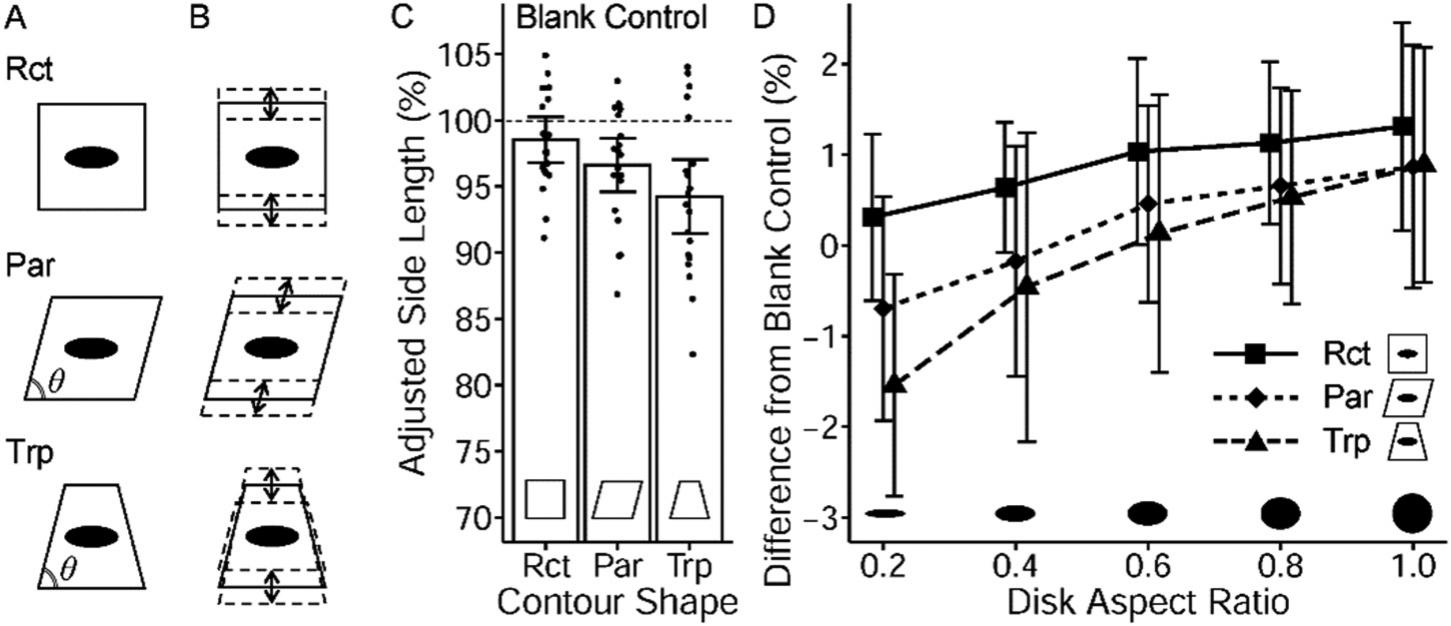
(A) Experiment 4 presented a contour shape with a single black distorted disk (aspect ratio 0.2–1.0). The contour shape was either a rectangle (Rct), parallelogram (Par), or trapezium (Trp). For Par and Trp, the angle *θ* was 75°. (B) The task was to adjust the side length so that it seemed equal to the base length of the contour. (C) The mean adjusted side length (%) relative to the base length for the blank-control condition wherein contour shapes were shown without the disk. Each dot represents the mean of each participant. (D) The results of contour shapes with a disk as a function of the disk aspect ratio. Differences from the corresponding blank-control conditions are shown. Error bars represent 95% CI.

The stimulus images comprised contour shapes and a black disk (Figure 5A). They were black and presented on a white background. The contour shape was either a rectangle (Rct), parallelogram (Par), or trapezium (Trp). The angles spanned by the side line and the bottom base line were 90° (Rct), 75° (Par, acute angle), and 75° (Trp). The length of the bottom base line was 12.4° (452 pixels), which was identical to that in Experiments 1–3.

A black disk was placed inside the contour shape. The disk aspect ratio was varied among 0.2 (strongly foreshortened), 0.4, 0.6, 0.8, and 1.0 (circle). The width of the disk was maintained at 6.2° (226 pixels). Additionally, there were blank-control conditions wherein the disk was not presented. Consequently, 18 stimulus conditions were selected.

Blank-control conditions were used to separate the effect of the disks from that of contour shapes. As in Experiments 1–3, the effect of the disks was measured by subtracting the blank-control condition's adjusted side length from the adjusted side length with a disk.

#### Apparatus and Procedure

Each participant performed five trials for each of the 18 stimulus conditions, yielding 90. The order of the trials was randomized. A short break was provided after every 30 trials. Experiment 4 typically took 20–25 min.

Participants pressed the space key to start each trial, and a stimulus figure was presented on the 24-inch LCD screen. At the beginning of the trial, the side line length was randomly set between 67% and 133% of the base line. The center of the stimulus figure was randomly jittered from the screen center so that the participants could not compare the side line length across trials. The task was to adjust the side line so that its length looked equal to the bottom base line. Participants were explicitly informed in advance that the task was to adjust the length on the screen and not the length in the 3D space. Participants lengthened or shortened the side line by pressing the computer's up/down cursor key (Figure 5B). No time limitations were set for the task. The disk position was fixed throughout the trial. The bottom base length and the interior angles never changed. Thus, in the trapezium contour, adjusting the side line length changed the length of the top base. When the participants pressed the space key, the adjusted side length was recorded, and the subsequent trial was initiated.

### Results and Discussion

The adjusted side length is expressed as a percentage of the base length (100% indicated no illusory effect). Adjustments shorter than 100% indicated an overestimation of the side line length.

The mean adjusted side lengths of the blank control conditions are shown in Figure 5C. A one-way repeated-measures ANOVA revealed a significant main effect of contour shapes (*F*(2, 38) = 10.3, *p* < .001). Multiple *t*-tests with Shaffer-Bonferroni correction (α = 0.05) revealed that Rct yielded longer adjustments than Par and Trp, and Par yielded significantly longer adjustments than Trp. Even without the disk, the parallelogram and trapezium contours yield an illusory effect because those shapes suggest a slant. The effect was stronger for trapezium contours.

The effect of the disk aspect ratio was assessed by analyzing the difference in the adjusted length from the blank control. For each aspect ratio for each participant, the mean adjusted side length of the blank-control condition was subtracted from the mean adjusted side length of the condition with the disk in which the same contour shape was used. As illustrated in Figure 5D, the disk aspect ratio had an effect. A two-way repeated measures ANOVA (contour shape × disk aspect ratio) on this data revealed a significant main effect of the disk aspect ratio (*F*(4, 76)_ _= 5.9, *p* < .001). Multiple comparisons (Shaffer-Bonferroni correction, α = 0.05) indicated that a disk aspect ratio of 0.2 yielded significantly lower values than 0.6, 0.8, and 1.0. The main effect of contour shape and two-way interaction were not statistically significant (*F*(2, 38) = 1.9, *p* = .163; *F*(8, 152) = 0.6, *p* = .791, respectively). To summarize, irrespective of the contour shape, the disk with an aspect ratio of 0.2, which implied a large slant angle, showed an illusory effect in which the side length was perceived to be longer than the conditions with a larger disk aspect ratio.

## Experiments 5A and 5B

The purpose of Experiment 5 was to investigate the effect of the number of disks. Experiment 4 demonstrated that the disk aspect ratio influenced the perception of the side line length, which could explain the original illusion shown in Figure 1A. However, this illusion can also be explained by the number of disks. As seen in the Oppel-Kundt illusion and Helmholtz square (Robinson, 1998, pp. 49–52), a region filled with horizontal lines appears taller, and a region filled with vertical lines appears wider. Therefore, if the disks in Figure 1A played the role of these filling lines, the original illusion could be explained by the Oppel-Kundt illusion, not by the slant perception created by the disks. To address this issue, Experiment 5 determined whether the number of disks would influence the perceived side length.

### Method

#### Participants

Nineteen individuals (mean age = 23.1) participated in Experiment 5A, and another set of 19 individuals participated in Experiment 5B. Since the data of one participant was excluded due to technical difficulties during the experiment, the data of the remaining 18 participants (mean age 22.7 years) were analyzed in Experiment 5B. All participants reported normal or corrected-to-normal vision.

#### Stimuli and Procedure

The task and apparatus used were identical to those used in Experiment 4. Again, participants observed the stimuli on the computer screen and adjusted the side line length to look the same as the bottom base line length.

**Figure 6.**
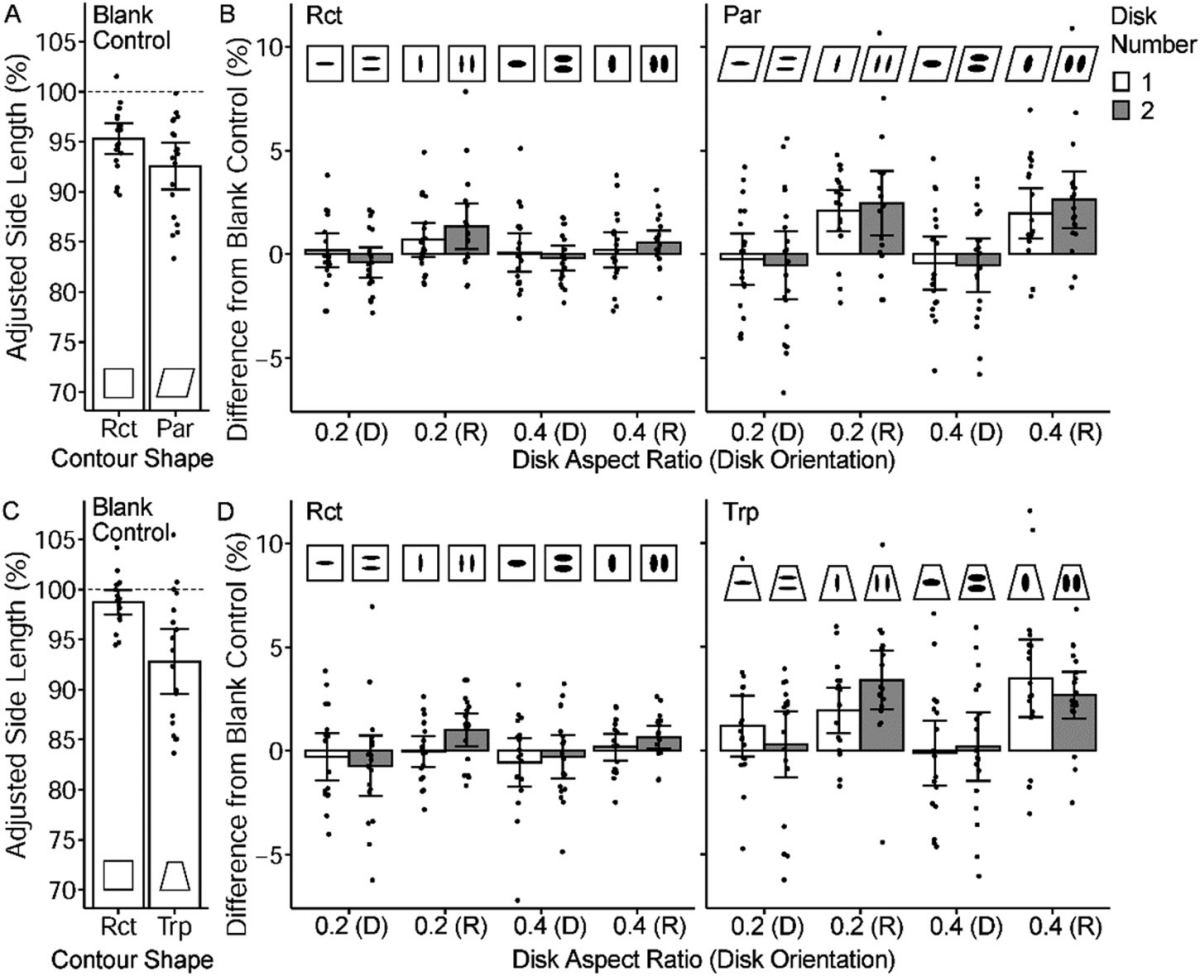
(A) and (B) indicate the results of Experiment 5A, while (C) and (D) indicate the results of Experiment 5B. The task assigned was identical to that of Experiment 4. One or two disks were shown, except for blank-control conditions. The disk aspect ratio was 0.2 or 0.4. The disks were shown with in-depth orientation (abbreviated as D in the figure) or rotated orientation (abbreviated as R). Error bars represent 95% CI. Each dot represents the mean of each participant.

In Experiment 5A, two contour shapes were adopted, Rct and Par. In the blank-control conditions, only the contour shapes were presented with no disks. Under other conditions, the disks were presented inside contour shapes. The disks were manipulated by disk number (1 and 2), disk aspect ratio (0.2 and 0.4), and disk orientation (depth and rotated). In combination with the two contour shapes, 16 non-control conditions were prepared (see Figure 6B). The disk orientation of depth indicated that each disk looked foreshortened in depth, as in Experiment 4. Each disk was rotated by 90° (Rct) or 75° (Par) in the rotated condition. Together with the two blank-control conditions, Experiment 5A comprised 18 conditions. Each participant performed 5 trials per condition, with 90 trials total, which took approximately 20–25 min.

Rct = rectangle; Par = parallelogram; Trp = trapezium.

Experiment 5B was identical to 5A, with two exceptions. The Trp contour shape replaced the Par contour shape. In the rotated conditions, the disks were rotated by 90° in all conditions. Figures 6C and D contain the entire list of stimuli used.

If the disks caused the Oppel-Kundt illusion, (i) the side length would be perceived as long and thus adjusted shorter for two disks than for one disk in the depth orientation, and (ii) the base line would be perceived as long, and thus the side length would be adjusted longer for two disks than for one disk in the rotated orientation.

### Results of Experiment 5A

The mean adjusted side length in Experiment 5A blank-control conditions was longer for Rct than for Par (paired *t*(18) = 3.86, *p* = .001, *d* = 0.59; Figure 6A), which was consistent with the results of Experiment 4 (Figure 5C). In addition, even for the Rct blank-control condition, the side length was perceived to be longer than the actual length, as reflected in the significantly shorter adjusted length than 100% (*M* = 95.3%, *t*(18) = 6.42, *p* < .001). This result was attributable to the classical horizontal-vertical illusion (Robinson, 1998, pp. 96–100; Wolfe et al., 2005).

As in Experiment 4, differences from the blank-control conditions (Figure 6B) were analyzed to test the effect of disk parameters. A four-way repeated-measures ANOVA (disk number × disk aspect ratio × disk orientation × contour shape) was conducted. Only disk orientation showed a significant main effect (*F*(1, 18) = 23.6, *p* < .001), while the other factors did not (disk number, *F*(1, 18) = 0.2, *p* = .633; disk aspect ratio, *F*(1, 18) = 1.9, *p* = .181; contour shape, *F*(1, 18) = 2.0, *p* = .178). However, disk orientation showed significant two-way interactions with disk number (*F*(1, 18) = 4.6, *p* = .045) and contour shape (*F*(1, 18) = 10.7, *p* = .004). The simple main effect of disk number was not significant for either depth orientation (*F*(1, 36) = 1.2, *p* = .284) or rotated orientation (*F*(1, 36) = 3.27, *p* = .079), indicating that the disk orientation × disk number interaction was not reliable. The simple main effect of contour shape was not significant for depth orientation (*F*(1, 36) = 0.4, *p* = .514), but was significant for rotated orientation (*F*(1, 36) = 8.9, *p* = .005). No other interaction was statistically significant (*p*s > .1).

### Results of Experiment 5B

The mean adjusted side length in Experiment 5B blank-control conditions was longer for Rct than for Trp (paired *t*(17)_ _= 3.93, *p* = .001, *d* = 1.13; Figure 6C), which was consistent with the results of Experiment 4 (Figure 5C). As well as in Experiment 5A, even the Rct blank-control condition yielded a significantly shorter adjusted length than 100% (*M* = 98.7%, *t*(17) = 2.23, *p* = .040), which was due to the horizontal-vertical illusion.

The difference from the blank-control conditions (Figure 6D) was analyzed using the same design of four-factor ANOVA in Experiment 5A. The main effects of disk orientation (*F*(1, 17) = 24.7, *p* < .001) and contour shape (*F*(1, 17) = 8.2, *p* = .011) were significant, but the disk number (*F*(1, 17) = 1.0, *p* = .337) and disk aspect ratio (*F*(1, 17) = 0.1, *p* = .737) did not show a significant main effect. Despite the absence of a main effect of disk number, the three-way interaction of disk number, disk aspect ratio, and disk orientation was significant (*F*(1, 17) = 10.2, *p* = .005). As reflected in this interaction, a simple main effect of disk number was significant only for disks with an aspect ratio of 0.2 in the rotated orientation (*p* = .005). In this condition, participants adjusted the side length to be longer for the two-disk figure than for the one-disk figure. This result may not indicate that the two rotated disks decreased the subjective side length. The two rotated disks were more likely to increase the subjective base length because of the Oppel-Kundt illusion. Since the task was to adjust the side line to look the same length as the base line, the participants adjusted the side line longer. The disk number did not have a significant effect for disks of 0.2 aspect ratio in depth orientation, and those of 0.4 aspect ratio (*p*s > .1).

The two-way interaction between the disk orientation and contour shape was significant (*F*(1, 17) = 18.8, *p* < .001). The simple main effect of contour shape was not significant for depth disk orientation (*F*(1, 34) = 2.0, *p* = .164), but was significant for rotated disk orientation (*F*(1, 34) = 16.3, *p* < .001). This interaction pattern was also observed in Experiment 5A. No other interactions were significant (*p*s > .07).

### Discussion

The results implied that the disks yielded Oppel-Kundt illusion in some conditions with rotated disks. However, the disk number did not affect the figures with depth disk orientation. Considering this result and the finding that even a single disk's in-depth orientation could cause an illusory perception of side length (Experiment 4), the illusion found in the original figure (Figure 1A) could not be attributable to the Oppel-Kundt illusion alone.

## General Discussion

The experimental results reported above suggest that the aspect ratio of the foreshortened disks was the primary source of the illusory effect observed in Figure 1A. A smaller aspect ratio, which suggested a larger slant angle, increased the perceived side line length (Experiment 4, Figure 5D). However, disk number yielded virtually no effect on the illusion and slant perception (Experiment 5). Furthermore, pictorial depth cues other than the disks were found to influence the perceived side line length, suggesting that those cues were also integrated into the slant perception of the object surface.

### Effect of the Surface Features

Experiments 1B and 2B (3D length estimation) showed that the disks and the textures yielded 3D slant perception. Such slant perception was likely to yield an illusorily longer perception of 2D side length (Experiments 1A and 2A). This effect suggested that the perceived slant by surface features is integrated into the metric perception of 3D object shape in an automatic and mandatory way. Although trapezium contour shapes without surface features served as depth cue as well, they yielded slant underestimation (blank-control conditions, Experiments 1B, 2B; Saunders & Backus, 2006a; Smith, 1967). The strongly foreshortened disks would have offset (partly) such underestimation. The disks outside the object did not affect the perceived side line length of the object (Experiment 3), further supporting the conclusion that the disks yielded the illusory perception of side line length via slant perception of the object surface.

Similar interactions between the perception of object contour shapes (or frames) and surface figures embedded in a contour have been previously reported. For example, when a frame of a slanted screen (i.e., trapezium contour in the retinal image) is visible, it partially contributes to the perceptual constancy of shapes shown on the screen (Vishwanath et al., 2005; Wallach & Marshall, 1986). Likewise, Sander's illusion has been interpreted as the result of improper perceptual constancy caused by the parallelogram contour, although such an explanation has been questioned (Robinson, 1998, pp. 24–26). In any case, these previous studies have shown that trapezium/parallelogram contours suggest a slant and influence the shape perception of figures embedded in the contour. In contrast, the present study demonstrated an effect of opposite direction: figures inside the contours alter the perception of contour shapes.

Though weaker than the disks, texture gradients showed a similar effect (Experiment 2). The effect of texture orientation in Experiment 2A ranged from 0.33 to 2.00 (in the unit of dependent variable), while it was larger (2.37–3.40) in Experiment 1A using disks. Previous works on slant perception using textured rectangles also demonstrated the little or modest effect of texture gradients (Saunders & Backus, 2006a; Todd & Norman, 2003; Zimmerman et al., 1995). The regular texture yielded more effect than the irregular texture, which was consistent with the previous findings showing that regular textures contain more depth information and thus contribute to the more salient, veridical perception of slant (Kraft & Winnick, 1967; Newman et al., 1973; Saunders & Backus, 2006b; Velisavljević & Elder, 2006). Grid or checkerboard textures may be more effective than the “regular” texture used in the present study, as they appear to have more information on a slant (see Anderson et al., 1998; Todd et al., 2005). However, Saunders and Backus (2006a) reported only a minimal (but reliable) effect of adding a checkerboard texture on slant perception.

### Effect of Disk Number and Oppel-Kundt Illusion

Although two disks aligned side-by-side might widen the perceived object shape in some (but not all) conditions, two vertically aligned disks did not increase the perceived side line length compared to the single-disk figure (Experiment 5, Figure 6). The former effect may be attributable to the Oppel-Kundt illusion (Robinson, 1998, pp. 49–52) rather than slant perception. A typical Oppel-Kundt figure uses tick lines for the filling elements, while the illusion also occurs with dots (Bertulis et al., 2009).

However, it seemed doubtful that the stimuli with two disks yielded the Oppel-Kundt illusion because the illusion weakens for stimuli with few filling elements (<5) and even reverses (i.e., underestimation of length) for stimuli filled with one element (Bertulis et al., 2009; Coren & Girgus, 1978, p. 27; Mikellidou & Thompson, 2014; Rentschler et al., 1981). Taken together, it is unlikely that the illusory effect shown in Figure 1A is wholly attributable to the Oppel-Kundt illusion.

### Effects of the Object Contours

It was also evident that the disks were not the only factor that affected the perceived side line length. The contour shape of the upper surface of the object has an illusory effect. Even without the disks, trapezium/parallelogram contour shapes yielded illusorily longer perception of their side lines compared to the rectangle (Experiment 4, Figure 5C; Experiments 5A and 5B, Figures 6A and 6C). As described in the introduction, trapezium/parallelogram shapes yield slant perception, albeit often underestimated (Saunders & Backus, 2006a; Smith, 1967). Indeed, the results of 3D length estimation for blank-control stimuli (Experiments 1B and 2B) implied slant underestimation, as the estimates were below the veridical length of 100. Adding disks or textures of depth-orientation condition, which indicated a larger slant angle, might decrease this slant underestimation. In addition, Experiment 4 showed a less powerful illusory effect for parallelograms than trapeziums (Figure 5C). This result was likely due to the absence of convergence cues in parallelograms.

Rectangle contours without disks yielded an illusory, longer perception of their side lines in Experiment 5, although this effect did not have statistical significance in Experiment 4. This illusory overestimation of rectangular side length (i.e., height) was minor compared to the side line overestimations for trapezium/parallelogram contours and would be the horizontal-vertical illusion (Robinson, 1998, pp. 96–100). The horizontal-vertical illusion is well known for the “T” figure, but the “L” figure still yields the illusion (Finger & Spelt, 1947; Wolfe et al., 2005). It is also known that the illusion effect decreases for “∠” figures with acute angle (Wolfe et al., 2005). In the present study, however, the trapezium with 75° angle yielded a more pronounced overestimation of side length compared to the rectangle with 90° (Experiment 4, Figure 5C), further supporting the conclusion that the overestimation of side length in trapezium/parallelogram contours was due to depth/slant perception rather than the horizontal-vertical illusion.

Another intriguing finding on the contours was the difference between the box and surface conditions. Even without the disks/textures, the box-like object increased the perceived side line length compared to the planar surface object (Experiment 1A, Figure 2D; Experiment 2A, Figure 3D; Experiment 3, Figure 4A). The 3D length estimation results also suggested that the box object yielded the perception of a larger (i.e., more veridical) slant angle of the upper surface (Figures 2F and 3F). The side surface of the box object might provide additional perspective clues to veridical slant perception if observers assumed that the side surface and the upper surface were at 90°. Alternatively, the side surface may have added a context to reinforce the 3D interpretation of the stimulus images. A comparable phenomenon can be found in the Shepard illusion (Shepard, 1981); its illusory effect seems stronger for box-like or table-like figures than for parallelogram figures (i.e., tabletop only).

### Conclusions

**Figure 7.**
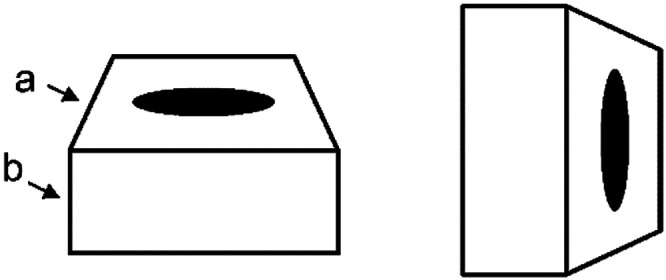
Illusory length perception by surface figures and contours. The side line length (a) looks longer than the length of line (b), despite the fact that they are equal in length. Three factors influenced the perceived side length (a): aspect ratio of the foreshortened surface figure, contour shape (trapezium in this figure) of the upper surface of the object, and the rectangular side surface attached to the upper surface. The illusory effect seems unchanged when the figure is rotated 90° (right figure).

By examining the illusory effect of the side line length of the upper surface, the present study demonstrated that multiple depth cues from surface features and contour shapes are integrated for slant perception from 2D images. The illusory effect was maximum for a strongly foreshortened disk (aspect ratio 0.2), trapezium contour, and box object. Figure 7 demonstrates their effects; the side line (a) appears longer than another side line (b).

Foreshortened surface figures seemed more effective than texture gradients for slant perception, while figures outside the target object were ineffective. Contours of the side faces of the box object provided additional depth cues that were integrated into the slant perception of the upper surface of the object. Pictorial depth cues belonging to the target object were integrated for slant perception, but cues outside the object might not be. This implication seems consistent with the recent theory of stereopsis (Vishwanath, 2014), which posits a distinction between the perception of relative depth among objects and the perception of absolute, egocentric depth.
